# Volume tracking – a novel method for visualization and quantification of intracardiac blood flow from 3D time resolved phase contrast MRI

**DOI:** 10.1186/1532-429X-11-S1-P129

**Published:** 2009-01-28

**Authors:** Johannes Töger, Martin Ugander, Håkan Arheden, Einar Heiberg

**Affiliations:** Cardiac MR Group, Dept. of Clinical Physiology, Lund, Sweden

**Keywords:** Particle Trace, Arbitrary Shape, Trace Technique, Dimensional Flow, Cardiac Blood

## Introduction

Three-dimensional time resolved flow measurement may provide greater insights into cardiovascular dynamics, as the detailed interactions of blood, myocardium, valves and vessels are not completely understood. Since three-dimensional flow data is highly complex, better methods providing both an intuitive visualization and quantification are needed. Existing methods have focused on tracking particles. However, following a volume may be more intuitive and easier to interpret visually. More importantly, it may also enable quantification of physical parameters of the tracked volume, which may provide novel physiological insight.

## Purpose

The purpose of the study was to develop and test the feasibility of Volume Tracking, a new method for visualization and quantification of three-dimensional intracardiac blood flow. Specifically, we sought to develop a method which offers the possibility of tracking volumes, as opposed to previous methods where only particles can be tracked.

## Methods

Four healthy volunteers underwent acquisition of three-dimensional time resolved phase contrast velocity MRI data using a Philips 3 T scanner. State-of-the-art numerical methods were used to solve a novel representation of fluid transport which implicitly follows blood flow through the heart. Visualization was undertaken using the software Ensight (CEI, USA).

## Results

**Figure 1:**
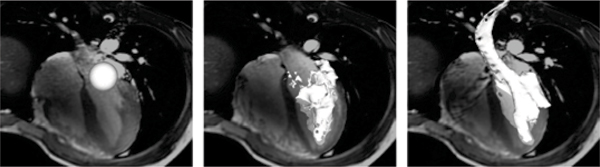
**An example of Volume Tracking visualization in a healthy volunteer**. Left: Early diastole, a white spherical volume of blood positioned near the mitral annulus. Middle: Late diastole, the same blood volume has now deformed as it has entered the left ventricle. Right: Early systole, additional deformation of the volume as it is ejected into the aorta.

**Figure 2:**
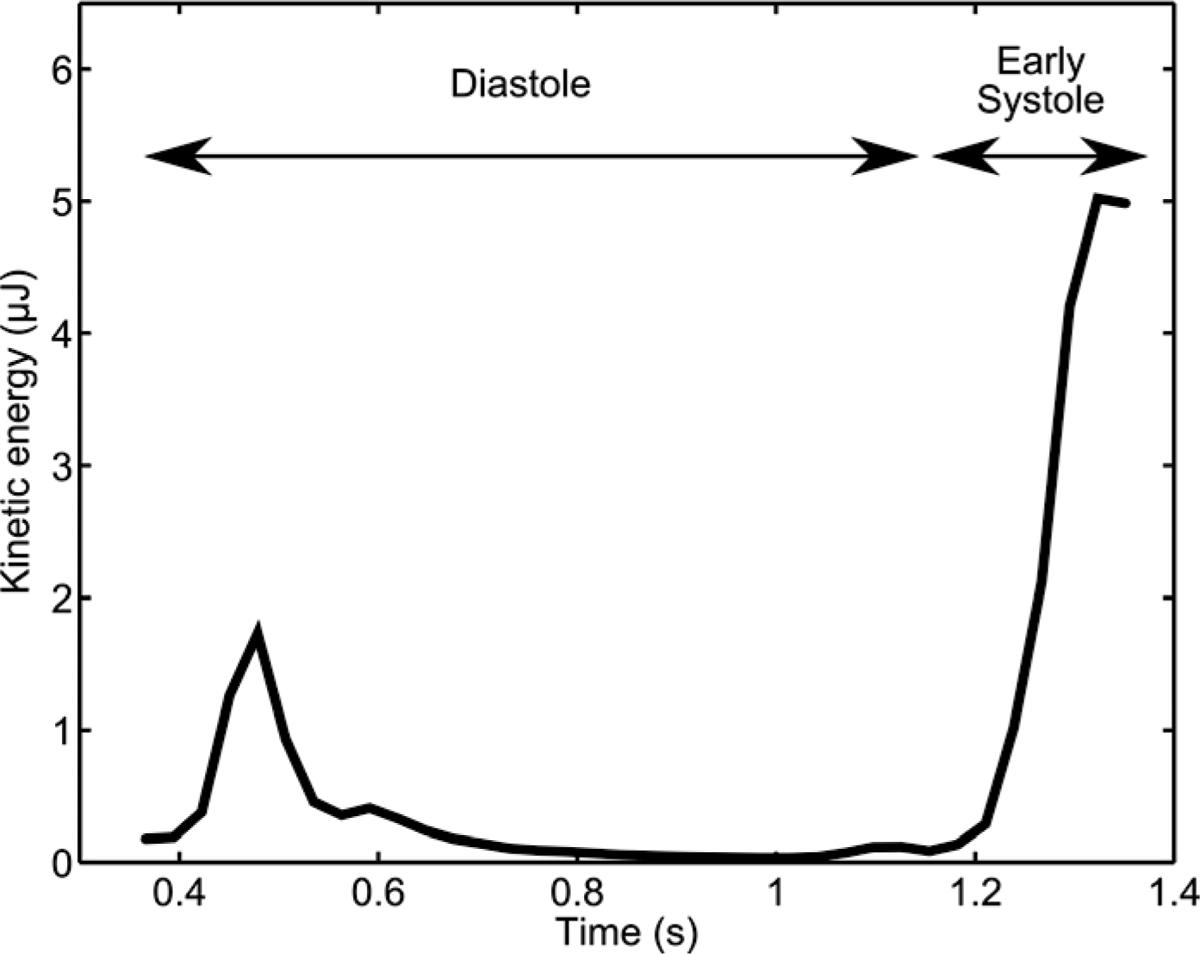
**Results for quantification of the kinetic energy over time for the tracked volume from Figure 1**. Note the slight increase in kinetic energy in the volume during early diastole and the sizeable increase in kinetic energy during early systole.

The proposed Volume Tracking method is feasible. Volumes of arbitrary shapes and sizes can be interactively visualized and tracked over the cardiac cycle. Intuitive visualization (Figure 1) and quantification of physical parameters such as kinetic energy (Figure 2) was achieved.

## Conclusion

A new method for visualization and quantification of intracardiac blood flow has been developed. The major advantage compared to the existing particle trace technique is the ability to quantify kinetic energy, momentum and other physical parameters of the blood flow. The method also offers real time interactivity and intuitive visualization of volumes moving through the heart. Once the data has been computed, exploring volumes of arbitrary shapes and sizes is straightforward. This new quantification and visualization method may facilitate the analysis of three dimensional flow and may bring additional physiological insight into cardiac blood flow.

